# Prophylactic treatment with PEGylated bovine IFNλ3 effectively bridges the gap in vaccine-induced immunity against FMD in cattle

**DOI:** 10.3389/fmicb.2024.1360397

**Published:** 2024-04-04

**Authors:** Sarah E. Attreed, Christina Silva, Monica Rodriguez-Calzada, Aishwarya Mogulothu, Sophia Abbott, Paul Azzinaro, Peter Canning, Lillian Skidmore, Jay Nelson, Nick Knudsen, Gisselle N. Medina, Teresa de los Santos, Fayna Díaz-San Segundo

**Affiliations:** ^1^Plum Island Animal Disease Center, Plains Area, Agricultural Research Service, U.S. Department of Agriculture, Greenport, NY, United States; ^2^Oak Ridge Institute for Science and Education Plum Island Animal Disease Center Research Participation Program, Oak Ridge, TN, United States; ^3^Department of Pathobiology and Veterinary Science, University of Connecticut, Storrs, CT, United States; ^4^Animal Biosciences and Biotechnology Laboratory, Northeast Area, Agricultural Research Service, U.S. Department of Agriculture, Beltsville, MD, United States; ^5^VetBio Partners, LLC., Carmel, IN, United States; ^6^Ambrx Biopharma, Inc., La Jolla, CA, United States; ^7^National Bio-and Agro-Defense Facility, Plains Area, Agricultural Research Service, U.S. Department of Agriculture, Manhattan, KS, United States; ^8^Office of Biodefense, Research Resources and Translational Research, National Institute of Allergy and Infectious Disease, Rockville, MD, United States

**Keywords:** FMDV, foot-and-mouth disease, type III interferon, IFN, IFNλ3, IL28B, PEGylation, biotherapeutics

## Abstract

Foot-and-mouth disease (FMD) is a vesicular disease of cloven-hoofed animals with devastating economic implications. The current FMD vaccine, routinely used in enzootic countries, requires at least 7 days to induce protection. However, FMD vaccination is typically not recommended for use in non-enzootic areas, underscoring the need to develop new fast-acting therapies for FMD control during outbreaks. Interferons (IFNs) are among the immune system’s first line of defense against viral infections. Bovine type III IFN delivered by a replication defective adenovirus (Ad) vector has effectively blocked FMD in cattle. However, the limited duration of protection—usually only 1–3 days post-treatment (dpt)—diminishes its utility as a field therapeutic. Here, we test whether polyethylene glycosylation (PEGylation) of recombinant bovine IFNλ3 (PEGboIFNλ3) can extend the duration of IFN-induced prevention of FMDV infection in both vaccinated and unvaccinated cattle. We treated groups of heifers with PEGboIFNλ3 alone or in combination with an adenovirus-based FMD O1Manisa vaccine (Adt-O1M) at either 3 or 5 days prior to challenge with homologous wild type FMDV. We found that pre-treatment with PEGboIFNλ3 was highly effective at preventing clinical FMD when administered at either time point, with or without co-administration of Adt-O1M vaccine. PEGboIFNλ3 protein was detectable systemically for >10 days and antiviral activity for 4 days following administration. Furthermore, in combination with Adt-O1M vaccine, we observed a strong induction of FMDV-specific IFNγ+ T cell response, demonstrating its adjuvanticity when co-administered with a vaccine. Our results demonstrate the promise of this modified IFN as a pre-exposure prophylactic therapy for use in emergency outbreak scenarios.

## 1 Introduction

Foot-and-mouth disease (FMD) is one of the most economically devastating agricultural illnesses globally. The etiologic agent of this disease is FMD virus (FMDV), a positive sense, single stranded RNA virus of the family *Picornaviridae*. FMDV is the most rapidly replicating virus known. Its incubation period is short, levels of viral shedding are exceptionally high—particularly in pigs—and the rate of transmission between affected animals is rapid (Grubman and Baxt, 2004). There are seven FMDV serotypes [A, O, C, Asia 1 and South African Territories (SAT) 1, 2, and 3] and many subtypes (reviewed in Domingo et al., 2002 and Grubman and Baxt, 2004). Clinical disease is characterized by fever, depression, anorexia, lameness, salivation, and development of vesicular lesions on the hooves, mouth, snout, and teats of cloven-hoofed animals. International trade of FMDV-susceptible animals or derived products from enzootic countries is prohibited by international policies (World Organisation for Animal Health, 2015). This has significant economic impacts for countries with ongoing outbreaks and is a significant biosecurity risk to FMD-free countries. The currently approved chemically inactivated whole virus FMD vaccine (Doel, 2003), and an adenoviral vectored subunit FMD vaccine (Ad5-FMDV) approved for emergency use in the United States (Grubman et al., 2010; Moraes et al., 2011), take at least 7 days to confer protection to vaccinated animals, during which time they are still susceptible to infection. There is a significant focus on developing biotherapeutics (Medina et al., 2020b; Kim et al., 2022) or antiviral agents (Li et al., 2019; Mei-Jiao et al., 2019; Naeem et al., 2021; Zhang et al., 2023) to prevent the occurrence of FMDV infections during the vulnerable period prior to vaccine-induced humoral immunity, thereby closing this window of susceptibility.

Interferons (IFNs) are among the first line of defense against viral pathogens. During a typical viral infection, common pathogen-associated molecular patterns (PAMPs) bind cellular pattern recognition receptors (PRRs) in infected host cells and trigger signaling cascades, leading to the upregulation and secretion of Types I and III IFNs (reviewed in Lazear et al., 2019, Mesev et al., 2019, and Carty et al., 2021). Interaction of these IFNs with their cellular receptors triggers signal transduction cascades that initiate the upregulation of a battery of IFN stimulated genes (ISGs) responsible for limiting viral replication through a variety of mechanisms (reviewed in Schoggins, 2019). As a result of these selective pressures, viruses develop adaptations to evade host immune responses. Certainly, FMDV is one of the most notorious immune-evading viruses (reviewed in Medina et al., 2018). It earned this reputation through its ability to: shut down cap-dependent translation; cleave important innate immune signaling proteins; induce multiple cellular membrane rearrangements; upregulate autophagy; and more (Devaney et al., 1988; Monaghan et al., 2004; Teterina et al., 2006; de Los Santos et al., 2007; Odonnell et al., 2011; Wang et al., 2011a; Gladue et al., 2012). Over 20 years of FMDV research has demonstrated how acutely sensitive the virus is to pre-exposure prophylactic treatment with Types I, II and III IFNs. In the early 2000s, Chinsangaram et al. demonstrated that treatment of bovine cells with recombinant IFNα or IFNβ—two type I IFNs—suppressed FMDV replication at the level of translation (Chinsangaram et al., 1999, 2001). Similar results were observed in porcine cells and, most importantly, in swine pre-treated with a replication defective human adenoviral 5 (Ad5)-vector expressing the porcine IFNα gene (Chinsangaram et al., 2003). Moraes et al. (2007) found that treating with an Ad5-vectored porcine IFNγ—the only member of the type II IFN family—in combination with the Ad5-vectored porcine IFNα, at 1 day prior to challenge with FMDV, induced enhanced antiviral activity and fully protected swine against disease. Similar results were observed in mice by using a single Ad5 vector co-expressing porcine IFNs-α and-γ (Kim et al., 2014). Recombinant porcine IFNδ8 and ovine IFNζ, both members of the type I IFN family, have also been shown to effectively upregulate ISGs and protect against two FMDV serotypes in cells (Usharani et al., 2017; Li et al., 2018). However, treatment of cattle with Ad5-vectored type I or type II IFN only had limited efficacy (Wu et al., 2003).

Type III IFNs, the latest family of IFNs described, have also been a subject of study for application as a prophylactic treatment against FMD. Currently there are three subtypes of bovine type III IFN also known as IFNλs or interleukin (IL) 28/29: IFNλ1 or IL29; IFNλ2 or IL28A; and IFNλ3 or IL28B (Kotenko et al., 2003; Sheppard et al., 2003). We have previously demonstrated that bovine and porcine IFNλ3 effectively block FMDV infection *in vitro* and *in vivo* in cattle and swine. One attractive characteristic of IFNλ is that—due to the tissue restriction of its receptor (a heterodimer of IL-28Rα and IL-10Rβ) to epithelial cells—there is low potential for off-target effects and inflammatory pathology related to the therapeutic itself (Diaz-San Segundo et al., 2011). In 2011, replication of FMDV was shown to be inhibited by treatment with an Ad5-vector system-secreted IFNλ3 in bovine EBK and MDBK cells (Diaz-San Segundo et al., 2011) or by treatment with recombinant porcine IFNλ1 in porcine IBRS-2 cells (Wang et al., 2011b). Moreover, systemic antiviral activity and induction of ISGs were detected in cattle treated with the Ad5-vectored bovine IFNλ3 (Ad5-boIFNλ3) (Diaz-San Segundo et al., 2011, 2016). Perez-Martin et al. (2012) demonstrated that inoculation of cattle with Ad5-boIFNλ3 significantly upregulated selected ISGs in the upper airways, protecting them against FMDV challenge. The same was demonstrated in swine using a similar Ad5porIFNλ3 (Perez-Martin et al., 2014). In 2016, the Ad5-boIFNλ3 platform was used in combination with an Ad5-FMDV O1Manisa vaccine in cattle, inducing a strong CD4 and CD8 IFNγ response within 2 days of treatment. Interestingly, the combination was 100% effective at preventing clinical disease in cattle challenged with FMDV at 3 days post-vaccination, despite a lack of detectable neutralizing antibody response at that time, suggesting Ad5-boIFNλ3 might have an adjuvant effect on cellular immunity (Diaz-San Segundo et al., 2016). The combination treatment also more robustly upregulated the expression of key immune receptors, including CD40L, CD80/CD86, and CCR7, which play crucial roles in promoting adaptive immune responses and memory T cell polarization.

Still, however, the therapeutic window for application of these antiviral treatments is limited and the production costs of the adenovirus vectors as a method of delivery are high. To overcome the relatively limited half-life of these IFN prophylactic treatments *in vivo* and reduce the cost-per-dose, synthesis of recombinant proteins with modifications such as polyethylene glycol conjugation (PEGylation), immunoglobulin Fc fragment or albumin fusion, among others, have been applied to some of the most promising IFN prophylactic therapies, including porcine IFNα (Miyakawa et al., 2011; Vallee et al., 2012; Lazear et al., 2019; Diaz-San Segundo et al., 2021). It is well known that PEGylation can be used to modulate the biophysical properties and/or biological activity of a biotherapeutic protein. Indeed, Diaz-San Segundo et al. (2021) used a pegylated porcine IFNα (PEGpoIFNα) as a successful strategy to prevent clinical FMD in swine challenged 5 days post treatment. However, high doses of protein were required, causing pleiotropic side effects (i.e., jaundice) in some cases, likely due to the ubiquitous distribution of the Type I IFN receptor.

Typically, PEG moieties are covalently linked to a target biotherapeutic via its naturally occurring amino acid residues, such as lysine or cysteine, or the N-terminus, which contain reactive moieties. However, the reactive sites of these naturally-occurring amino acids, which may seem suitable for PEGylation, may play a significant role in receptor binding. Thus, indiscriminate attachment of polymer chains such as PEG to such reactive sites on a biotherapeutic protein can lead to a significant reduction or even total loss of its biological activity (Clark et al., 1996). PEG derivatives can also undergo side reactions with residues other than those targeted for modification, which can create complex and poorly defined heterogeneous mixtures of PEG-derivatized biotherapeutics with reduced biological activity. One technology which promises to overcome many of these limitations is the incorporation of synthetic amino acids into proteins (see, e.g., Wang et al., 2001; Wang and Schultz, 2002; Chin et al., 2003; Tian et al., 2014). These and other studies have demonstrated that it is possible to site-specifically introduce into a protein a synthetic amino acid containing a chemical functional group that is not found in the 20 common amino acids. These synthetic amino acids can be used to react efficiently and selectively form stable covalent linkages with moieties, such as water-soluble polymer moieties, that are chosen for conjugation with the protein.

The objectives of the current study were: to determine whether a novel, site-specific PEGylated boIFNλ3 (PEGboIFNλ3) could extend its half-life in cattle; to determine if administration of the PEGboIFNλ3 alone or in combination with an FMD vaccine prior to FMDV exposure could provide improved efficacy prior to the onset of protective antibody titers; and to determine whether PEGboIFNλ3 could act as a vaccine adjuvant when administered in combination with an FMD vaccine. Our results demonstrate that this molecule exhibits extended biological activity and that it fully protects against FMDV challenge in cattle, both alone and in combination with an FMD vaccine, when administered 3–5 days prior to challenge. PEGboIFNλ3, therefore, shows promise as both a biotherapeutic and adjuvant capable of both effectively bridging the immunity gap following vaccination and boosting adaptive immunity against FMD in cattle.

## 2 Materials and methods

### 2.1 Cells and viruses

HEK 293 cells (ATCC CRL-1573) were used to generate and propagate recombinant Ad5 vectored FMD vaccine (Adt-O1M). LF-BK cells (LaRocco et al., 2013) were used for propagation and titration of FMDV serotype O1Manisa and for assessing serum neutralizing antibody titers. BHK-21, clone 13 (ATCC CCL-10) were used to propagate FMDV SAT 1 and to measure virus titers by end point titration. MDBK cells (ATCC CCL22) were used for *in vitro* antiviral activity assays and for propagating vesicular stomatitis virus (VSV). MDBK-t2 (Fray et al., 2001) were kindly provided by B. Charleston (Institute for Animal Health, Pirbright, United Kingdom). HEK293, BHK-21, and MDBK-t2 cells were maintained in minimum essential medium (MEM) containing either 10% calf serum or 10% fetal bovine serum (FBS) supplemented with antibiotics, glutamine, and non-essential amino acids. MDBK-t2 media was further supplemented with 10 μg/mL blasticidin (Invitrogen, Carlsbad, CA, United States). MDBK and LF-BK cells were maintained in Dulbecco’s MEM (DMEM) supplemented with 10% calf serum or FBS, antibiotics, glutamine, and non-essential amino acids.

The vaccine virus Adt-O1M was produced as described elsewhere (Diaz-San Segundo et al., 2016). VSV NJ was provided by the Foreign Animal Disease Diagnostic Laboratory (FADDL) at Plum Island Animal Disease Center (PIADC), Greenport, NY, United States. The challenge virus FMDV O1Manisa (O1M) was produced from a natural derived isolate (Pacheco et al., 2016). Median bovine infectious dose (BID_50_) was determined in bovines by intradermal inoculation in the tongue of multiple dilutions (Henderson, 1952). FMDV O1M titers were determined by standard plaque assay on LF-BK cells. A FMDV SAT 1 field strain was provided by FADDL. FMDV RNA levels were determined using quantitative real time PCR and the AgPath-ID One-Step RT-PCR kit [Applied Biosystems (Waltham, MA, United States)].

Cultured cell monolayers were infected with FMDV as described elsewhere (Diaz-San Segundo et al., 2021).

### 2.2 Generation of PEGylated bovine IFNλ3

Recombinant boIFNλ3 was generated using the Ambrx *E. coli* expression system engineered for site-specific incorporation of synthetic amino acids (SAA) into protein sequences (see e.g., WO2006068802A2 and WO2007/021297). Using this system, boIFNλ3 was expressed with synthetic amino acid, p-acetyl-L-phenylalanine (pAcF), incorporated at amino acid site T119 to facilitate site-specific PEGylation. The T119 site was selected as a preferred PEGylation site for boIFNλ3 after screening multiple sites and was found to have improved antiviral activity, biophysical characteristics, and pharmacokinetic profile compared to other site variants. The recombinant boIFNλ3, expressed into inclusion bodies, was isolated, refolded, and purified to homogeneity. Following purification, boIFNλ3 was site-specifically conjugated at the T119pAcF site with a single aminooxy functionalized 30 kDa polyethylene glycol (PEG) molecule through a stable oxime bond. PEGylated boIFNλ3 was further purified to remove excess reagents from the conjugation reaction, formulated and characterized.

### 2.3 *In vitro* antiviral activity assay

Biological antiviral activity of recombinant boIFNλ3 and PEGboIFNλ3 was evaluated *in vitro* against gold standard VSV. Briefly, MDBK-t2 cells were treated with 2-fold dilutions of boIFNλ3 or PEGboIFNλ3 and incubated overnight at 37°C and 5% CO_2_. 24 h post treatment (hpt), cells were challenged with VSV NJ at MOI of 0.1 and incubated for 48 h. Titers of VSV were evaluated by TCID_50_ using a colorimetric MTT assay (Millipore Sigma, Burlington, MA, United States) following manufactures directions. Comparison of the antiviral activity of recombinant boIFNλ3 and PEGboIFNλ3 was also assayed against FMDV. Briefly, MDBK cells were treated at 2-fold dilutions of boIFNλ3 or PEGboIFNλ3 and incubated overnight at 37°C and 5% CO_2_. Cells were challenged with FMDV at MOI of 0.1 at 24 hpt and incubated for another 48 h at 37°C and 5% CO_2._ Titers of FMDV were evaluated in the cell supernatants by end point dilution on BHK-21 cells.

### 2.4 Animal experiments

The pharmacokinetics study was conducted at HMS Veterinary Development (Tulare, CA), in compliance with the Animal Welfare Act (AWA), 2020 and other laws and regulations governing the humane care of animals. The guidelines set forth by the Guide for the Care and Use of Agricultural Animals in Research and Teaching (Fourth Edition, 2020), were reviewed for pen stocking density. The efficacy study was performed in the high-containment facilities of the Plum Island Animal Disease Center (Greenpoint, NY, United States) in compliance with: the AWA; Guide for the Care and Use of Laboratory Animals; the 2002 Public Health Service Policy for the Humane Care and Use of Laboratory Animals; U.S. Government Principles for Utilization and Care of Vertebrate Animals Used in Testing, Research and Training (IRAC, 1985); as well as specific animal protocols reviewed and approved by the Institutional Animal Care and Use Committee (IACUC) of the Plum Island Animal Disease Center (USDA/APHIS/AC Certificate number: 21-F-0001; Protocol 244.01-19-R).

#### 2.4.1 Pharmacokinetics animal study

The pharmacokinetic study used eight 4–6 months old Holstein-Fresian calves, four males and four females equally divided in two groups. Calves were administered one subcutaneous injection of either 75 or 150 μg/kg PEGboIFNλ3 in the prescapular region of the neck. Sera was collected at the following times: pre-treatment, 0.5, 1, 3, 6, 12, 24, 36, 48, 72, 96, 120, 168, 240, and 336 hpt and analyzed for concentrations of PEGboIFNλ3 and systemic antiviral activity.

#### 2.4.2 Efficacy animal study

The study used a total of 18 Holstein heifer calves of about 450 lbs each (4–6 months old) and was performed to evaluate the efficacy of PEGboIFNλ3 *in vivo*, alone or in combination with Adt-O1M to prevent FMD. Six groups of three animals were subcutaneously (SQ) inoculated in the neck (inoculum divided equally between the right and left side of the neck) with 2.5 × 10^9^ pfu of either Adt-O1M or a mock Ad5-Blue, alone or in combination with 150 μg/kg PEGboIFNλ3, either 3 or 5 days prior to challenge. On the day of challenge, all cattle were exposed to 2 × 10^6^ BID_50_ of FMDV O1M administered intranasopharyngeally as previously described (Pacheco et al., 2016) and disease progression was followed for 3 weeks after challenge. The six treatment groups were as follows: Adt-O1M in combination with PEGboIFNλ3 at 5 days pre-challenge [PEGboIFNλ3 + Ad-O1M(−5dpc)]; Adt-O1M in combination with PEGboIFNλ3 at 3 days pre-challenge [PEGboIFNλ3 + Ad-O1M(−3dpc)]; Ad5-Blue in combination with PEGboIFNλ3 at 5 days pre-challenge [PEGboIFNλ3(−5dpc)]; Ad5-Blue in combination with PEGboIFNλ3 at 3 days pre-challenge [PEGboIFNλ3(−3dpc)]; Adt-O1M administered alone at 3 days pre-challenge [Ad-O1M(−3dpc)]; and Ad5-Blue administered alone at 3 days pre-challenge [Control]. One animal from the PEGboIFNλ3(−5dpc) treatment group moved during the injection and did not receive the full dose of biotherapeutic. The data generated from this animal were therefore removed from all analyses but can be found in Supplementary Table 1.

After challenge, animals were clinically examined daily and scored every other day until 8 days post-challenge (dpc). Clinical scores were determined by the number of feet presenting FMD vesicular lesions plus the presence of vesicles in the mouth/snout. The maximum score is 5. Rectal temperature data was monitored daily throughout the experimental period. Serum was collected daily between vaccination/treatment and 9 dpc to assess antiviral activity initially prior to challenge and viremia after challenge. Further serum samples were collected at the time of treatment, 0, 4, 7, 14, and 21 dpc, inactivated at 56°C for 30 min, and stored at −70°C to be used in a neutralization assay on LF-BK cells. Heparinized blood was collected and PBMCs purified at the time of inoculation and 0, 3, 7, 14, and 21 dpc to study the cellular immune response and analyze gene induction in leukocytes. Virus shedding was assessed daily from 0 to 9 dpc in nasal secretions. Complete blood count (CBC) was analyzed daily from 0 to 9 dpc in a Hemavet® 950 analyzer (Drew Scientific, Waterbury, CT, United States) to monitor lymphocytes using EDTA blood sample.

### 2.5 PEGylated bovine-IFNλ pharmacokinetic assay

Serum samples collected during the pharmacokinetic (PK) animal study were assayed at Ambrx Biopharma, Inc. for concentration of PEGboIFNλ3 by an electro-chemiluminescent assay (ECLA) using the Meso Scale Discovery (MSD) platform (Rockville, MD, United States). Briefly, MSD High Bind plates (MSD, L15XB) were coated with an anti-PEG capture antibody (Academia Sinica Cat# AGP4-PABM-A, RRID:AB_3075411) to discriminate PEGboIFNλ3 from endogenous IFNλ. The next day, plates were washed and blocked. Standards, QCs and study samples were diluted in bovine calf serum and added to the plates. A rabbit polyclonal anti-IL28B antibody (Sino Biological Cat# 11890-RP02, RRID:AB_3075410) was the primary detection reagent, and secondary detection consisted of SULFO-TAG labeled goat anti-rabbit antibody (Meso Scale Discovery Cat# R32AB, RRID:AB_2892814). Plates were read on an MSD QuickPlex SQ 120 reader after read buffer was added. The assay lower limit of quantitation was 2.49 ng/mL.

### 2.6 Antiviral biological assay in serum

MxCAT ELISA was used to determine units of antiviral activity of PEGboIFNλ3 as previously described (Diaz-San Segundo et al., 2013) using MDBK-t2 cells and a commercially available CAT-ELISA kit (Roche Applied Sciences, Indianapolis, IN, United States) in accordance with the manufacturer’s protocol. Units of antiviral activity per mL were calculated from the human IFNα2A standard curve.

### 2.7 Detection of virus in sera and nasal secretion

Cattle sera and nasal secretions were examined for the presence of virus by plaque assays on BHK-21 cells. Virus titers were expressed as log_10_ pfu/mL of serum or nasal swab secretions. The minimal detection level for this assay is 5 pfu/mL. In addition, FMDV RNA was detected by real-time quantitative PCR (RT-qPCR) as previously described (Alejo et al., 2013). Cycle threshold (Ct) values were converted to RNA copies per mL of serum or nasal secretion (Callahan et al., 2002).

### 2.8 Analysis of IFN stimulated genes and adaptive immune genes in PBMCs

IFN Stimulated Gene (ISG) expression in peripheral blood mononuclear cells (PBMCs) was analyzed by RT-qPCR as previously described (Diaz-San Segundo et al., 2016). Samples were run in an AB 7500 system (Applied Biosystems, Carlsbad, CA, United States) or in a QuantStudio 6 Flex (Applied Biosystems, Carlsbad, CA, United States). Relative quantification was performed on a panel of ISGs or adaptive immune genes as previously described (Diaz-San Segundo et al., 2016). The expression of each gene of interest was normalized using glyceraldehyde3-phosphate dehydrogenase (GAPDH). Data were analyzed using the comparative threshold cycle (ΔΔCT) method relative to baseline levels detected prior to treatment (Livak and Schmittgen, 2001).

### 2.9 Evaluation of humoral immune response

Serum neutralizing antibody titers (SNTs) were determined in cattle sera samples by end-point titration according to the Spearman-Kärber method (Oie, 2012). Antibody titers were expressed as the log_10_ value of the reciprocal of the dilution that neutralized 100 Tissue Culture Infectious Dose in 50% of the wells (TCID_50_) (Diaz-San Segundo et al., 2010).

### 2.10 Flow cytometric analysis of PBMCs

Peripheral blood mononuclear cells were isolated by density gradient centrifugation, red blood cells were lysed, and purified PBMCs were counted on a Vi-Cell Blu (Beckman Coulter, Brea, CA) and plated in triplicate at a density of 10^6^ PBMCs/well in 96-well round-bottom plates. PBMCs were stimulated as described elsewhere (Medina et al., 2015) with either FMDV O1M at MOI 2 or a general lymphocyte stimulant. Cells were labeled with LIVE/DEAD Fixable yellow viability dye (Invitrogen, Waltham, MA, United States), before staining with the following extracellular antibodies: mouse anti-bovine CD4-FITC (Bio-Rad Cat# MCA1653F, RRID:AB_321270), mouse anti-bovine CD3-PE-Texas Red (Bio-Rad Cat# MCA6080, RRID:AB_3075408, conjugated in-house using Abcam Cat# ab269899), mouse anti-bovine WC1-PerCPcy5.5 (Bio-Rad Cat# MCA1655, RRID:AB_1222696, conjugated in-house using Abcam Cat# ab102911), mouse anti-CD8-AlexaFluor-647 (Bio-Rad Cat# MCA837A647, RRID:AB_2275821), and mouse anti-bovine CD335-APCcy7 (Bio-Rad Cat# MCA2365, RRID:AB_2149298, conjugated in-house with Abcam Cat# ab102859). Cells were then fixed, permeabilized using BD’s Fixation/Permeabilization Kit (Cat# 554714) and the BD Permeabilization 2 Buffer (Cat# 340973) and intracellularly stained with mouse anti-bovine IFNγ-RPE (Bio-Rad Cat# MCA1783PE, RRID:AB_324003). Data expressed as the difference in percent of the single positive T cell parent population between the stimulated and unstimulated wells. All plates were run on an Agilent NovoCyte 3000 (violet, blue and red lasers) with NovoSampler Pro System and data were analyzed in NovoExpress Software version 1.5.0.

### 2.11 Data analyses

For the analysis of PEGboIFNλ3 PK results, data reduction and analysis was performed with MSD Discovery Workbench 4.0 and MS Excel software. PK parameters were calculated using noncompartmental analysis in Phoenix WinNonlin version 8.3.1 software. All other parameters were assessed by repeated measures one-way ANOVA within treatment group, with follow-up comparisons of each timepoint compared against either the day of treatment or the time-matched Control group by Fisher’s Least Significant Difference Test.

## 3 Results

### 3.1 Site-specific PEGylation of boIFNλ3 does not significantly affect its biological potency

**Figure 1:**
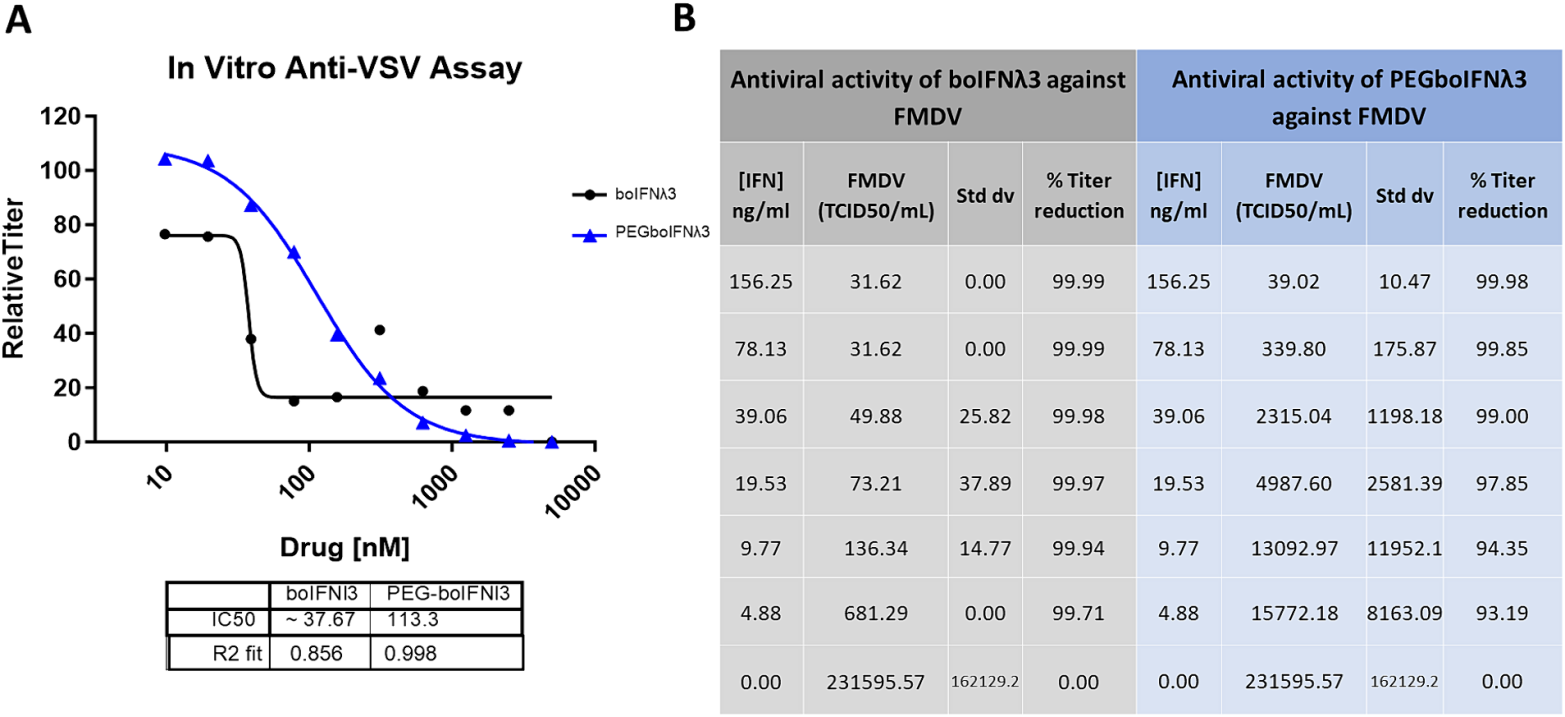
**(A)** Virus yield reduction assay of recombinant boIFNλ3 or PEGboIFNλ3 against VSV NJ. MDBK-t2 cells were treated with 2-fold dilutions of boIFNλ3 or PEGboIFNλ3. After overnight incubation, cells were challenged with VSV NJ at MOI of 0.1 and incubated for 48 h. Titers of VSV were evaluated by TCID_50_ and expressed as relative titer as compared to untreated cells. Average data points from duplicate measurements are represented. A sigmoidal dose–response curve was fitted to determine IC_50_ values for each recombinant IFN. **(B)** *In vitro* antiviral activity of recombinant boIFNλ3 vs. recombinant PEGboIFNλ3 against FMDV SAT1. MDBK cells were treated with 2-fold dilutions of boIFNλ3 or PEGboIFNλ3. After overnight incubation, cells were challenged with FMDV SAT1 at MOI of 0.1 and incubated for another 48 h_._ Titers of FMDV were evaluated in the cell supernatants by end point dilution on BHK-21 cells.

We have previously demonstrated that boIFNλ3 expressed using the replication-defective human Ad5 vector platform effectively blocks FMDV replication *in vitro* (Diaz-San Segundo et al., 2011). In this study, we aimed at testing the antiviral activity of boIFNλ3 when delivered as a PEGylated protein. Since traditional PEGylation can influence the binding affinity of therapeutic proteins to cellular receptors and, therefore, affect their bioactivity (Harris et al., 2001), several boIFNλ3 muteins with synthetic amino acid para-acetyl-L-phenylalanine (pAF) site-specifically incorporated into select positions of boIFNλ3 protein were designed, recombinantly produced, and subsequently PEGylated to determine whether the bovine IFNλ3-pAF muteins or their PEGylated counterparts would retain antiviral activity. Bovine IFNλ3 site-specifically PEGylated at position T119 via a stable oxime linkage with pAF was selected for further evaluation, and its antiviral activity against VSV and FMDV was compared against a non-PEGylated protein *in vitro*. Our results demonstrate that although site-specific PEGylation slightly reduced the antiviral activity *in vitro* against gold standard VSV NJ as compared to non-PEGylated recombinant boIFNλ3 protein (3-fold reduction in IC_50_; Figure 1A), the reduction was minor considering the potential for an increased half-life. Similar results were observed when antiviral activity was tested against FMDV (Figure 1B).

### 3.2 Circulating PEGboIFNλ3 and systemic antiviral activity against FMDV is prolonged *in vivo* after a single dose

**Figure 2:**
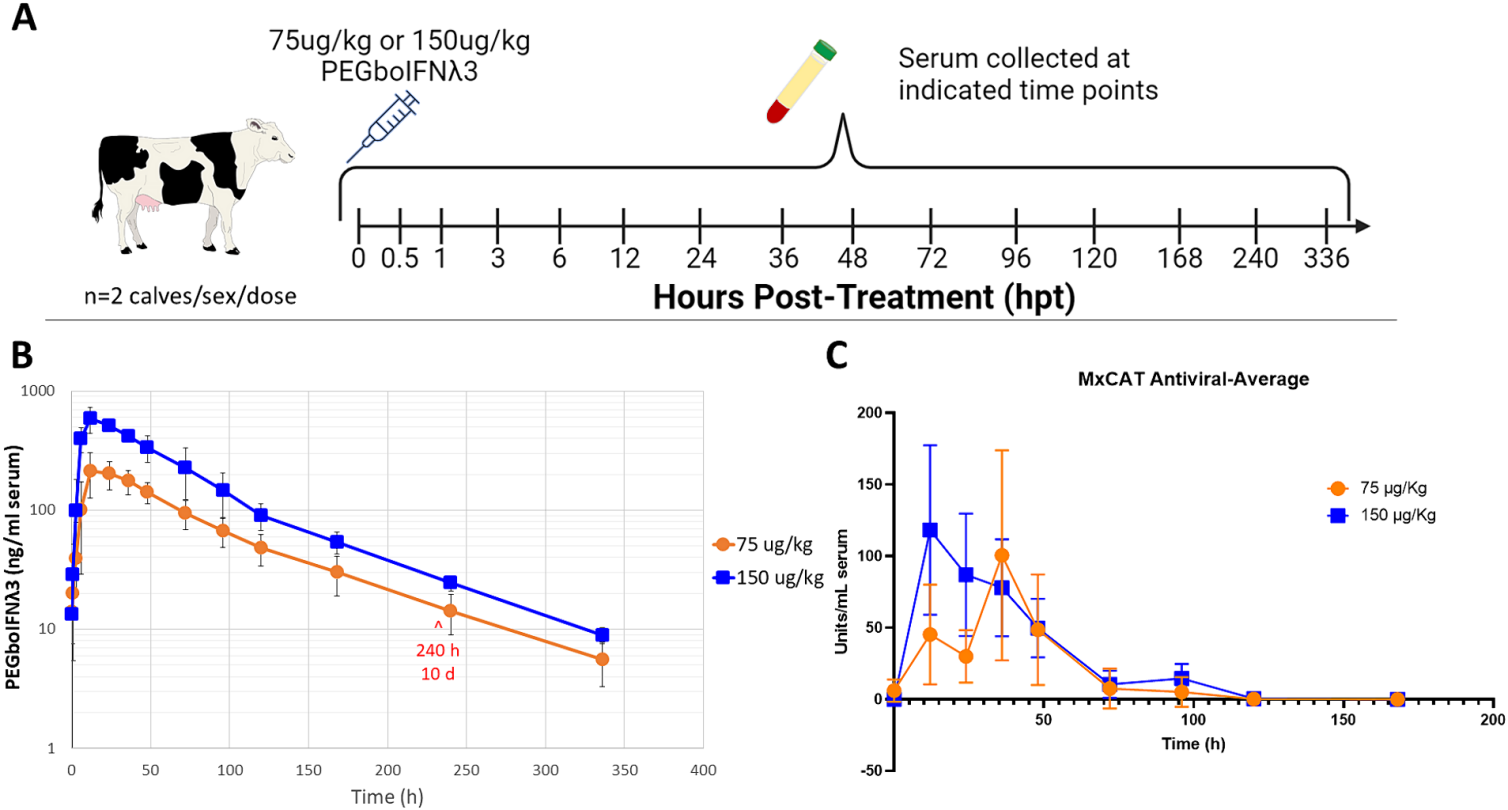
**(A)** Four-to-six-month-old Holstein-Fresian calves were injected with 75 or 150 μg/kg PEGboIFNλ3 and blood was collected at various time points for pharmacokinetic analysis. **(B)** PEGboIFNλ3 concentration in the serum was measured on the Mesoscale Discovery (MSD) platform via an electrochemiluminescent assay (ECLA). **(C)** Serum antiviral activity was measured via Mx CAT ELISA on cattle serum from the pharmacokinetic study. *n* = 2 cattle/sex/dose.

To test the pharmacokinetics of PEGboIFNλ3, groups of four Holstein-Fresian calves (two males and two females) were inoculated with either 75 or 150 μg/kg of PEGboIFNλ3 (Figure 2A). Serum concentration of PEGboIFNλ3 peaked at 12 h post-treatment (hpt) for both dose groups, with mean Cmax and exposure (AUC) approximately 2.7- and 2.3-fold higher, respectively, for the 150 vs. 75 μg/kg dose group (Figure 2B; Table 1). The terminal half-life, 65 or 69 h, was similar for the two dose groups (Table 1).

**Table 1:** Individual and mean pharmacokinetics parameters for PEGboIFNλ3 in bovine serum following SQ administration.

Group	Animal	Sex	*R*^2^	Half-life (h)	Tmax (h)	Cmax [ng/mL]	AUClast [h*ng/mL]	AUCinf [h*ng/mL]	AUC %Extrap
Group 1 (75 μg/kg)	1	M	0.999	72.8	36	177	18,471	19,322	4.4
	4	M	1.000	74.7	12	150	12,449	12,842	3.1
	6	F	1.000	60.9	12	272	18,319	18,646	1.8
	8	F	1.000	68.4	12	304	23,697	24,358	2.7
	Avg			69.2		226	18,234	18,792	
	%CV			8.8		33	25	25	
Group 2 (150 μg/kg)	2	M	0.990	69.7	24	522	36,504	37,265	2.0
	3	M	1.000	63.4	12	535	51,156	51,905	1.4
	5	F	0.999	62.8	12	635	39,494	40,463	2.4
	7	F	0.993	65.4	12	759	39,965	40,838	2.1
	Avg			65.3		613	41,780	42,618	
	%CV			4.8		18	15	15	

Serum samples from the same groups of Holstein-Fresian calves were assessed via Mx CAT ELISA for upregulation of IFNα2a as a measure of antiviral activity. Antiviral activity peaked earlier among cattle in the 150 μg/kg treatment group at 12 and 36 hpt among the 75 μg/kg dose treatment group (Figure 2C). Antiviral activity returned to baseline around 5 days post-treatment. Comparison of these results with previously published data from animals inoculated with Ad5-boIFNλ3 (Perez-Martin et al., 2012), indicates that PEGylation of boIFNλ3 induces longer-lived systemic antiviral activity in cattle.

### 3.3 Pre-treatment with PEGboIFNλ3 induces a protective antiviral state against FMDV infection in cattle

**Figure 3:**
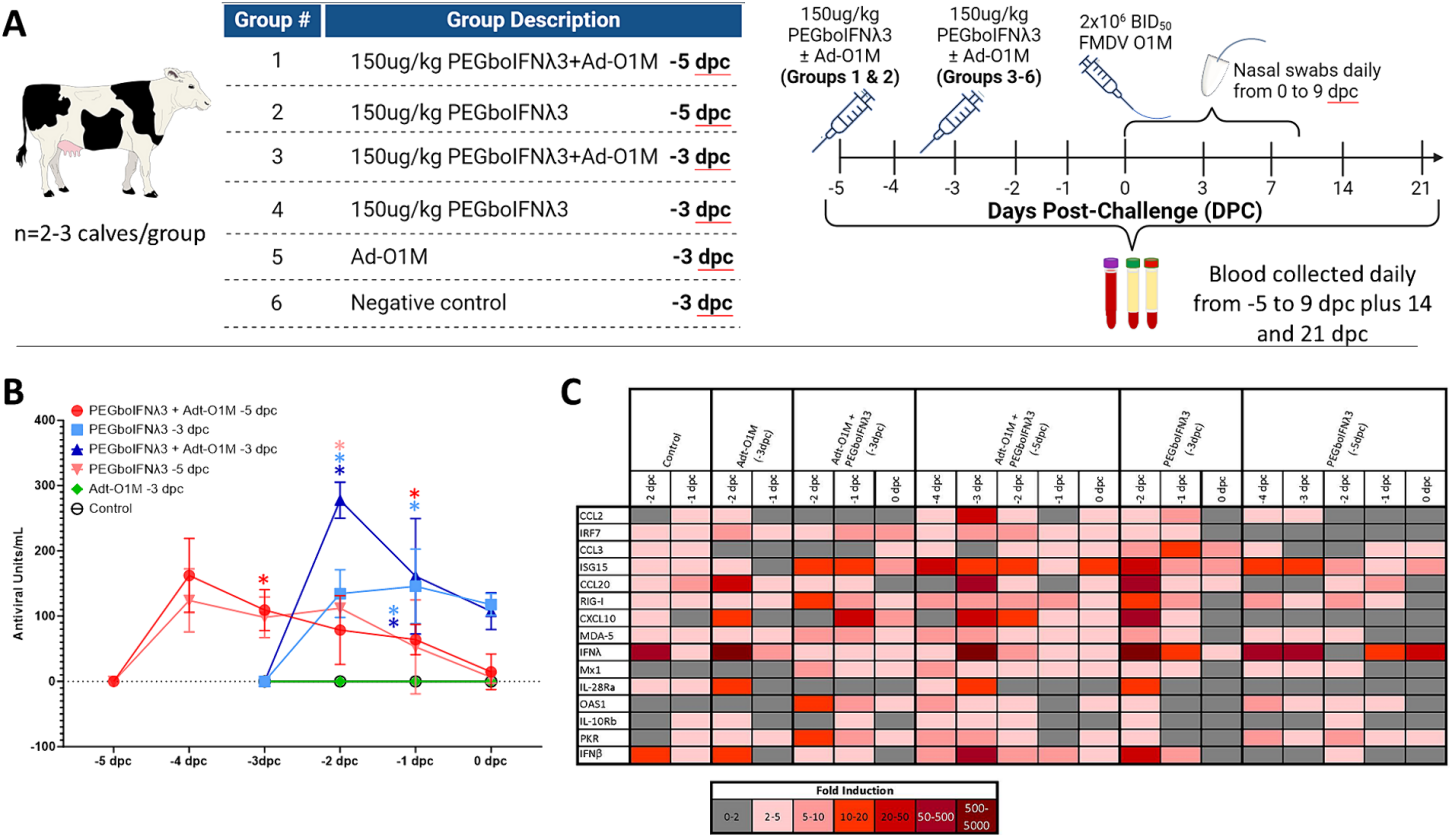
**(A)** Holstein calves of approximately 450 lb were subcutaneously injected with 150 μg/kg PEGboIFNλ3 and/or 2.5 × 10^9^ pfu Adt-O1M FMD vaccine at either 3 or 5 days prior to intranasopharyngeal challenge with 2 × 10^6^ BID_50_ FMDV O1Manisa. A control group was inoculated at 3 days prior to challenge with 2.5 × 10^9^ pfu Ad5-Blue. Blood was collected daily after treatment and serum and purified peripheral blood mononuclear cells (PBMCs) were preserved for later testing. **(B)** Serum antiviral activity was assessed by Mx CAT ELISA. **(C)** Interferon stimulated gene (ISG) induction was assessed in purified PBMCs daily following treatment. Change in gene expression is represented as the mean fold induction of each gene compared to the baseline pre-treatment time point, shaded according to intensity of up-or down-regulation of the gene. *n* = 2–3 calves/treatment group/time point.

Efficacy of PEGboIFNλ3 to prevent clinical FMD in cattle was evaluated in a separate animal study. Based on the results of the PK study, groups of three Holstein heifers were SQ administered 150 μg/kg of the PEGboIFNλ3 either alone or co-administered with an Adt-O1M FMD vaccine, either 3 or 5 days prior to challenge with wild type FMDV O1 Manisa (Figure 3A). Serum samples were collected daily leading up to challenge and assessed for antiviral activity by Mx CAT ELISA. While serum antiviral activity among the two groups treated at-5 days post-challenge (dpc) with PEGboIFNλ3 with or without Adt-O1M reached baseline by the day of the challenge (0 dpc), serum antiviral activity remained high at 0 dpc among animals in the two groups treated with PEGboIFNλ3 at-3 dpc (Figure 3B). Animals inoculated with Adt-O1M alone, as well as control cattle, did not show detectable levels of antiviral activity.

To assess the potential strength and duration of the induction of ISGs (as reviewed in Williams, 1991; Schoggins, 2019) in the treated cattle, RT-qPCR on PBMCs harvested daily up to challenge was performed. The results show the strongest and most lasting induction of ISGs among animals co-administered PEGboIFNλ3 + Adt-O1M (−5 dpc) in all measured ISGs (Figure 3C). Despite upregulation in several genes in the animals from the control group, upregulation of genes was generally higher in the rest of the groups that received PEGboIFNλ3. While some ISGs were most strongly induced at 1 day following treatment (ISG15, Mx1, OAS1, PKR, RIG-I, and MDA-5), others were most strongly induced at 2 days following treatment (CCL2, CCL20, IFNλ, IL-28Ra, and IFNβ). For the most part, induction of ISGs was more long-lasting in the cattle receiving the combination treatment when compared against the time-matched PEGboIFNλ3 alone group.

**Figure 4:**
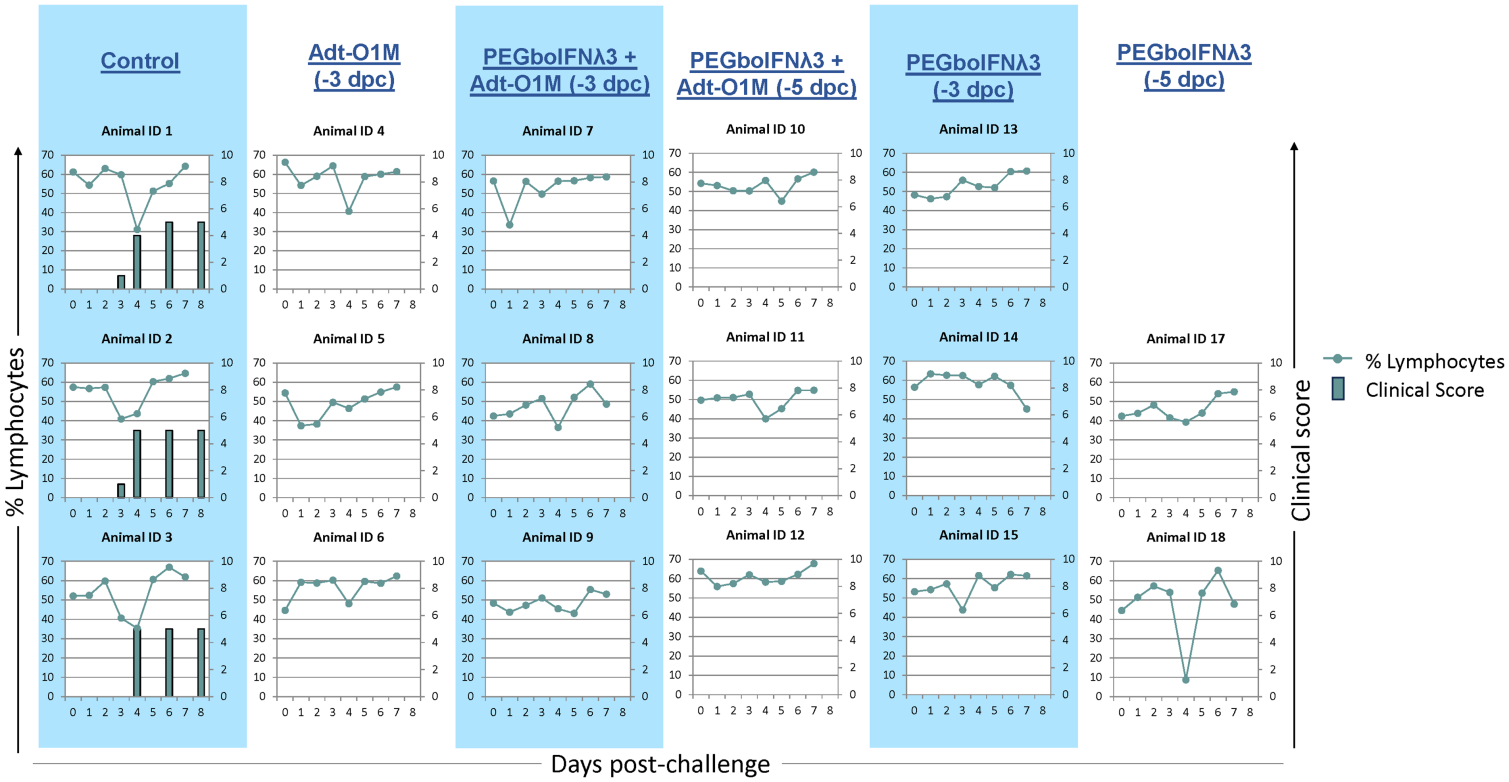
Holstein calves of approximately 450 lb were subcutaneously injected with 150 μg/kg PEGboIFNλ3 and/or 2.5 × 10^9^ pfu Adt-O1M FMD vaccine at either 3 or 5 days prior to intranasopharyngeal challenge with 2 × 10^6^ BID_50_ FMDV O1Manisa. A control group was inoculated 3 days prior to challenge with 2.5 × 10^9^ pfu Ad5-Blue. Cattle were assessed for clinical score (bars) on days 3, 4, 6, and 8 post-challenge and EDTA-treated blood was assessed for signs of lymphopenia daily (dotted line). *n* = 2–3 cattle/time point/treatment group.

As expected, all control animals began developing clinical vesicular disease between 3 and 4 dpc with FMDV O1Manisa, with a simultaneous peak in characteristic severe lymphopenia (Figure 4). Conversely, none of the cattle inoculated with either PEGboIFNλ3, Adt-O1M, or with the combination of the two, developed clinical symptoms (Figure 4). Interestingly, one heifer (Animal #18) experienced a severe, transient drop of over 40 percentage points in circulating lymphocytes at 4 dpc despite showing no clinical signs, viremia, or RNA-emia.

**Figure 5:**
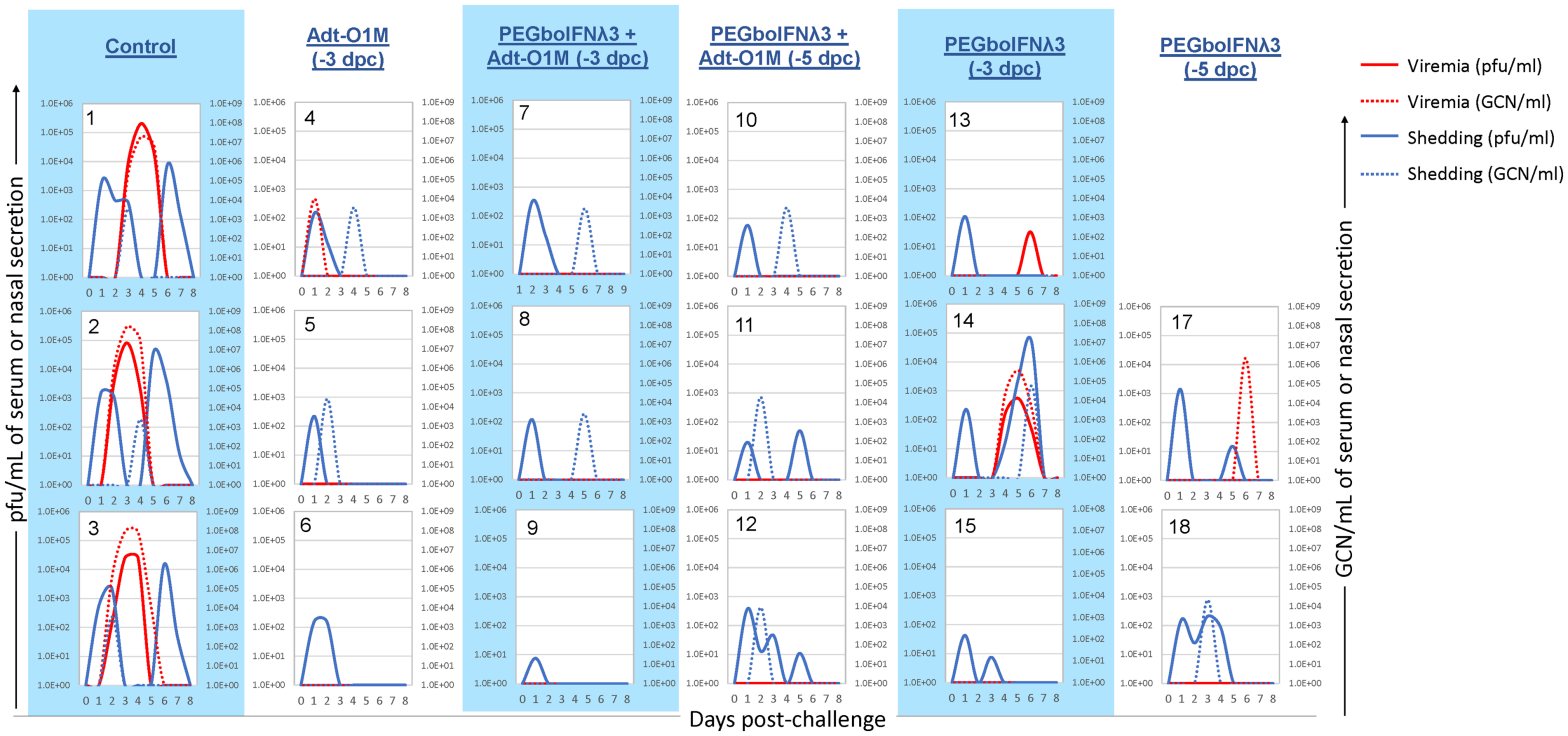
Holstein calves of approximately 450 lb were subcutaneously injected with 150 μg/kg PEGboIFNλ3 and/or 2.5 × 10^9^ pfu Adt-O1M FMD vaccine at either 3 or 5 days prior to intranasopharyngeal challenge with 2 × 10^6^ BID_50_ FMDV O1Manisa. A control group was inoculated 3 days prior to challenge with 2.5 × 10^9^ pfu Ad5-Blue. Daily, from 0 till 8 days post-challenge, serum and nasal swabs were collected and assessed for presence of FMDV. Viremia is reported in both PFU/mL of serum (solid red line) and GCN/mL of serum (dotted red line). Virus shedding is expressed in both PFU/mL of nasal secretions (solid blue line) and GCN/mL in nasal secretions (dotted blue line). *n* = 2–3 calves/treatment group/time point.

We next looked at virus dynamics in the nasal secretions and blood by both virus isolation and RT-qPCR. A transient peak of viral detection by virus isolation in the nasal secretion 1 day after challenge was observed in all animals (Figure 5), consistent with the route of challenge used, intranasopharyngeal (INP) inoculation, in which the virus was deposited in the nasopharyngeal cavity of the animal (Stenfeldt et al., 2015). Subsequently, control cattle showed consistent bimodal nasal shedding by virus isolation focused on days 1–2 and 5–6 post-challenge. Similarly, all animals in the control group showed virus by RT-qPCR in nasal secretion, although at a lower level and more temporally variable extent than by virus isolation. On the other hand, among the PEGboIFNλ3 treatment groups alone or in combination with Adt-O1M vaccine (−3 and-5 dpc), spikes of viral presence in nasal secretion were detected at lower titers/copy numbers than those seen in control animals, with the second peak in several animals being below the limit of detection by one or both methods. With respect to presence of systemic virus in the bloodstream, cattle in the control group consistently showed a peak of viremia by 3–4 dpc, by both virus isolation and RT-qPCR. Two out of three animals treated with PEGboIFNλ3 alone at −3 dpc showed much lower levels of viremia than control animals. In the cattle treated with PEGboIFNλ3 alone at 5 dpc or Adt-O1M at −3 dpc, only one in each group showed detectable RNA-emia, again at much lower levels than the control group. Interestingly, animals that received the combination treatment at either −3 or −5 dpc did not show any detectible viremia by either virus isolation or RT-qPCR.

### 3.4 Pre-treatment with PEGboIFNλ3 induces an adaptive immune response

**Figure 6:**
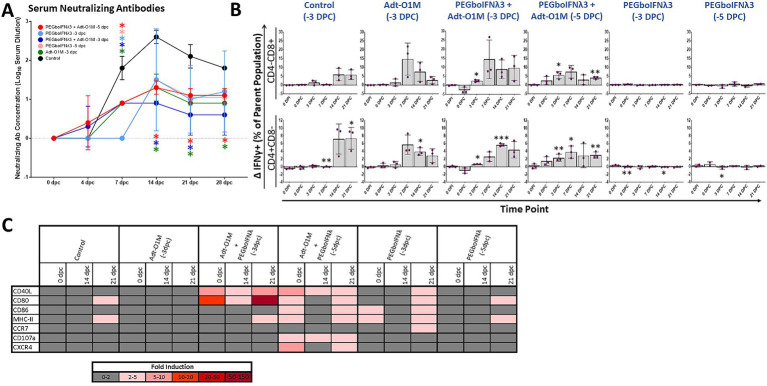
Holstein calves of approximately 450 lb were subcutaneously injected with 150 μg/kg PEGboIFNλ3 and/or 2.5 × 10^9^ pfu Adt-O1M FMD vaccine at either 3 or 5 days prior to intranasopharyngeal challenge with 2 × 10^6^ BID_50_ FMDV O1Manisa. A control group was inoculated 3 days prior to challenge with 2.5 × 10^9^ pfu Ad5-Blue. Blood was collected daily after treatment and challenge and serum and peripheral blood mononuclear cells (PBMCs) purified and preserved for later testing. *n* = 2–3 calves/treatment group/time point. **(A)** Heat-inactivated serum was tested at various time points for FMDV O1Manisa virus neutralizing titer. Titers expressed as the Log_10_ TCID_50_/mL of serum. ^*^p value < 0.05 compared to the control group at the given time-point. **(B)** Isolated PBMCs were stained for flow cytometric analysis. Upon *ex vivo* stimulation with MOI 2 FMDV O1Manisa, the induction of IFNγ expression in CD4-CD8+ and CD4 + CD8-T cell populations was measured and expressed as the difference in percent of the single positive T cell parent population between stimulated and unstimulated wells. **(C)** Adaptive immunity-related gene induction was assessed in PBMCs at various time points following challenge. Change in gene expression is represented as the mean fold induction of each gene compared to the baseline pre-treatment time point, shaded according to intensity of up-or down-regulation of the particular gene. ^*^p value < 0.05 compared to within-group 0 dpi ^**^p value < 0.01 compared to within-group 0 dpi ^***^p value < 0.001 compared to within-group 0 dpi.

Vaccine immunity against FMD is antibody-mediated (as reviewed in Doel, 2003). Therefore, we measured FMDV neutralizing antibody titers in serum at various time points following vaccination. By 0 dpc, none of our treatment groups had achieved a detectable level of anti-FMDV antibody (Figure 6A). Calves receiving both PEGboIFNλ3 and Adt-O1M vaccine (both −5 and −3 dpc treated) achieved a detectable level of anti-FMDV neutralizing antibodies at the earliest time point, 4 dpc, though all groups were quickly outpaced by serum antibody levels in the control group by 7 dpc. SNTs peaked in all groups at 14 dpc—apart from the PEGboIFNλ3 + Adt-O1M (−3 dpc) treatment group which peaked at 7 dpc—and remained steady till the end of the experiment. At 14 and 21 dpc SNTs among control animals remained significantly higher than both the Adt-O1M (−3 dpc) and PEGboIFNλ3 + AdtO1M (−3 and −5 dpc) treatment groups. By 28 dpc, the SNTs among control animals remained significantly higher than the Adt-O1M (−3 dpc) and PEGboIFNλ3 + AdtO1M (−5 dpc) groups.

Next, we assessed IFNγ production upon *ex vivo* specific restimulation in single positive CD4 or CD8 T cells as a proxy for virus specificity. Cattle in the combined treatment groups demonstrated significant IFNγ+ T cell responses earlier in the post-challenge period, by 3 dpc for both the −5 and −3 dpc combination therapy groups (Figure 6B). Conversely, there was no significant elevation in IFNγ+ T cells among groups administered PEGboIFNλ3 alone. Furthermore, the response at 3 dpc among the groups that received the combination treatment was more consistent compared to the vaccine alone group, which had one animal that did not respond (Figure 6B). Interestingly, we observed that both combination therapy groups displayed robust IFNγ+ CD4+ and CD8+ T cell populations at 0 dpc among unstimulated cells (percentages ranging from 1 to 5.5%). However, in cells incubated overnight with FMDV O1M, these IFNγ+ populations only increased in the PEGboIFNλ3 + Adt-O1M (−5 dpc) on the day of challenge, while they decreased among the PEGboIFNλ3 + Adt-O1M (−3 dpc) animals (data not shown). Importantly, T cells from these animals were strongly reactive to PMA stimulation (data not shown), indicating that any downregulation of IFNγ response was FMDV antigen-specific. At 7 dpc, the percentage of CD3-CD8 + CD335-cells positive for IFNγ expression was significantly increased over baseline among all animals that received Adt-O1M alone or in combination with PEGboIFNλ3 (Supplementary Figure 1A). While the cell surface characterization is incomplete, this population may represent a dendritic cell (DC) subset such as conventional DCs (Vremec et al., 2000; Schulz et al., 2002) and be involved in antigen cross-presentation with CD8+ T cells during viral infection (Belz et al., 2004). The percentage of NK cells (CD3-CD8 + CD335+) positive for IFNγ was elevated over baseline at 14 and 21 dpc among all vaccinated groups (Supplementary Figure 1B).

Finally, we assessed the expression levels of several genes involved in adaptive immunity in PBMCs over the course of the post-challenge period. Genes associated with DC antigen presentation functions (CD40L, CD80, CD86, and MHC-II) were most strongly upregulated at the time of challenge among groups receiving PEGboIFNλ3 either alone or in combination with Adt-O1M vaccine, though there was substantial variability across groups (Figure 6C). The most dramatic and sustained upregulation of CD40L and CD80 were observed among the Adt-O1M + PEGboIFNλ3 (−3 dpc) treatment group throughout the post-challenge period. These same genes were also consistently upregulated in the PEGboIFNλ3 + Adt-O1M treatment group (−5 dpc), while they were only upregulated at 21 dpc in the PEGboIFNλ3 (alone) treatment groups. Interestingly, upregulation of these genes among the control and Adt-O1M (−3 dpc) treatment groups was effectively nonexistent.

## 4 Discussion

Through site-specific PEGylation of boIFNλ3, we have demonstrated that we can: (1) achieve complete protection against FMD using recombinant PEGboIFNλ3 alone prophylactically; (2) extend the pre-exposure prophylactic window, effectively preventing clinical disease in FMDV-exposed cattle from 3 to 5 days pre-challenge; and (3) attain adjuvant effect of PEGboIFNλ3 when combined with an Ad5-FMDV vaccine, increasing the immunogenicity of the vaccine. These results highlight the exceptional versatility of PEGboIFNλ3 and its potential application during an emergency FMD outbreak response.

To the best of our knowledge, the present study is the first to report full clinical efficacy of an IFN therapy in cattle against FMD, within 3 and up to 5 days prior to challenge. Protection against clinical disease development among our PEGboIFNλ3 only treatment groups seems to be due largely to the extended antiviral activity afforded by site-specific PEGylation of boIFNλ3 via a stable oxime linkage to the synthetic amino acid pAF. Overall, similar clinical results were obtained in swine by treating them with large doses of PEGpoIFNα (Diaz-San Segundo et al., 2021), though this IFN only ever demonstrated partial protection when applied in cattle (Wu et al., 2003). Conversely, our previous Ad5-vectored boIFNλ3 study found that serum antiviral activity could only be detected until 2, but not 3 dpt (which was the day of challenge), consistent with the reduced protection observed among those animals administered the Ad5-boIFNλ3 treatment without concurrent FMD vaccine administration (Diaz-San Segundo et al., 2016). In the present study, we observed detectable antiviral activity in PEGboIFNλ3-treated cattle out to 4 days post-administration. Interestingly, we observed generation of serum neutralizing antibody levels in our PEGboIFNλ3 only treated animals comparable to those administered the combination therapy, indicating that this therapy does not prevent viral replication to a degree that would stop antibody formation (i.e., sterile protection). However, the induction of IFNγ+ T cells among PEGboIFNλ3 only treated cattle was suppressed in comparison to cattle given combination therapy. This data suggests that treatment with IFNλ3 therapy alone suppresses viral replication enough to shunt formation of a T cell response, while inducing an antibody response. However, further testing with greater sample sizes would be needed to support this hypothesis.

Previous studies from our lab have demonstrated tissue-specific upregulation of a variety of ISGs following treatment with an Ad5-boIFNλ3, particularly in the nasopharynx and palatine tonsil, and to a lesser extent in circulating PBMCs (Diaz-San Segundo et al., 2011). Given the kinetics of adenoviral vector clearance and the limited temporal range of translation of the boIFNλ3 gene within, the systemic antiviral activity afforded by this therapy was short-lived and animals that received only Ad5-boIFNλ3 all became clinically sick when challenged with FMDV at 3 dpt (Diaz-San Segundo et al., 2016). In the current study, while serum antiviral activity was reduced to near the limit of detection by 0 dpc in groups that were administered PEGboIFNλ3 at −5 dpc, a variety of ISGs were highly upregulated in circulating PBMCs in a sustained manner in all PEGboIFNλ3-treated groups. Among the most highly upregulated genes is ISG15, a potent antiviral (Perng and Lenschow, 2018). ISG15, a ubiquitin-like protein that serves a dual role in innate immunity, acts as both an intracellular protein modifier and an extracellular signaling molecule that boosts IFNγ secretion and has been reported to induce NK cell proliferation (D'Cunha et al., 1996), subsequently boosting the CD8+ CTL response (Iglesias-Guimarais et al., 2020), as we observe in our study in those cattle that were given combination therapies. Additionally, ISG15 induces DC cell maturation (Padovan et al., 2012), which may explain the upregulation of IFNγ in the assumed DC population CD3-CD8 + CD335-observed in this study. In a typical WT FMDV infection, Leader protease (L^pro^) inhibits several antiviral pathways in the host cell through cleavage of a variety of targets, including those modified by ISG15. However, our research group has previously demonstrated that overexpression of ISG15 in porcine cells can reduce WT FMDV replication *in vitro* (Medina et al., 2020a). This supports the idea that administration of PEGboIFNλ3 inhibits FMDV infection not only through direct antiviral mechanisms, but also by overwhelming the immune evasion strategies that FMDV employs, such as by upregulating ISG15. However, since we have only assessed expression at the transcript level, further studies would be needed to confirm protein levels and enzymatic activity. Other ISGs significantly upregulated by administration of PEGboIFNλ3 include RIG-I and MDA5, two members of the RIG-I-like receptor family of cytosolic RNA helicases that work by binding viral dsRNA. While MDA5 has been demonstrated to bind FMDV RNA, RIG-I has not, though this work was performed in porcine cells (Husser et al., 2011). PKR, Mx-1, and OAS1 were also shown to be upregulated in cattle that received the PEGboIFNλ3 treatment, consistent with past studies in this laboratory. These three genes are understood to play a role in the antiviral response against FMDV (de Los Santos et al., 2006), and while it has not been experimentally established that PKR interacts with FMDV RNA, depletion of PKR by siRNA or gene KO in tissue culture results in significantly higher virus yields (Chinsangaram et al., 2001; de Los Santos et al., 2006). Also, upregulated among PEGboIFNλ3-treated cattle were chemokines CXCL10 and CCL20, which have been shown to play a role in DC maturation, along with chemotaxis of DC and effector/memory T/B cells. Their expression in the context of FMDV vaccination and biotherapeutics is associated with protection against challenge (Diaz-San Segundo et al., 2010) and provides evidence of the adjuvanting effect of PEGboIFNλ3 when administered in conjunction with an FMD vaccine. Our results showed mild upregulation of several of the above-mentioned genes in the control group animals 1 or 2 days after inoculation with Ad5-Blue. Although this is somewhat surprising, it could be associated with the stress the animals were going through during manipulation for sample collection (Dhabhar, 2014), though more testing would be needed to confirm this hypothesis. However, importantly, the level of upregulation in the IFN-treated animals is consistently higher than the animals in the control group. Furthermore, the concerted and sustained systemic expression of these ISGs following treatment is consistent with the observed blockade of local and systemic viral replication among PEGboIFNλ3-treated cattle.

Previous literature demonstrates that FMD protective immunity is largely conferred by neutralizing antibodies and that this can occur in a T cell-dependent or-independent manner, depending upon whether the antigens are nonstructural or capsid-associated, respectively (Juleff et al., 2009; Carr et al., 2013). During the early post-vaccination period before neutralizing antibody titers are detected, strong innate immune activation and chemotaxis (Rigden et al., 2003), along with local antibody production may mediate immunity (Pega et al., 2013). This may serve as a partial explanation of immunity in the present study, even among the Adt-O1M treatment group, in light of the lack of antibodies and only low levels of IFNγ+ T cells. At the time of challenge, none of the cattle in the current study from any treatment group had detectable levels of circulating neutralizing antibodies, consistent with our 2016 study utilizing the Ad5-boIFNλ3. However, in that study, animals treated with both the vaccine and the Ad5-boIFNλ3 displayed an elevated percentage of IFNγ+ CD8+ and CD4+ T cells on the day of challenge. Moreover, the percentage of IFNγ+ CD8+ cells among animals administered only FMD Adt-O1M did not reach comparable levels to the combination treatment group until 5 dpc, indicating that the Ad5-boIFNλ3 may have acted as an adjuvant for the FMD Adt-O1M vaccine. In the current study, while robust T cell and neutralizing antibody responses developed in all cattle administered both PEGboIFNλ3 and Adt-O1M vaccine, this largely did not occur until after challenge. Importantly, animals receiving the combination treatment, Adt-O1M + PEGboIFNλ3, at either −3 or −5 dpc, developed significant levels of IFNγ+ T cells at 3 dpc. The group receiving vaccine alone demonstrated modest elevations in the levels of IFNγ+ T cells, but did not show significant upregulation in either of these cell populations until 14 dpc (CD4+). In general, the induction of IFNγ following restimulation was variable and even negative on certain days, despite small positive populations of these single-positive T cells among unstimulated cells (data not shown). This may be related to immune checkpoint pathways in cattle immunology. While there is a lack of research into T cell checkpoint controls in the context of FMDV, it has been established that cattle are capable of experiencing T cell dysregulation and exhaustion in the context of chronic or persistent infection (as reviewed in Konnai et al., 2017), as FMD often becomes in cattle. On a shorter time scale, checkpoint cell surface markers such as CTLA-4 (CD152) are shuttled to the immune synapse in pre-formed vesicles at a rate proportional to the strength of TCR stimulation and this increased cell surface presentation could lead to a dampening of IFNγ expression (Sansom et al., 2003; Watari et al., 2019). However, this deserves further exploration and clearly was not an impediment to the establishment of protective immunity in the current study.

Attending the T cell IFNγ response observed among cattle receiving the combination therapy in this study is an upregulation in several genes involved in adaptive immunity, most notably CD40L and CD80, though we found that expression even within groups was highly variable. The cell surface receptor CD40L is a costimulatory marker principally expressed on CD4+ T cells, which binds CD40 on DCs and B cells. The resulting signal transduction cascade increases survival and proliferation responses in both T and B cells, resulting in increased secretion of immunoglobulins from B cells (Hirano et al., 1997; Estes et al., 1998). Though susceptible to only an abortive infection, DCs experimentally infected with FMDV have been shown to downregulate CD40 expression, failing to stimulate T cell proliferation and leading to a dysfunctional T cell response early in FMDV infection (Ostrowski et al., 2005). While CD40 expression was not assessed in the current study, both the 2016 Ad5-boIFNλ3 study (Diaz-San Segundo et al., 2016) and the current study demonstrate that CD40L is significantly upregulated in a synergistic manner by the coadministration of PEGboIFNλ3 and Adt-O1M vaccine, boosting the T cell response. CD80 is a costimulatory molecule that is present on B cells and provides survival and activation signals to T cells (when bound to their CD28 receptor) and monocytes in a coregulatory partnership with CD86 (Dilioglou et al., 2003; Watari et al., 2019). Curiously, we did not see any upregulation of adaptive immunity genes at any of the time points tested among our Adt-O1M vaccine only treatment group, though cattle in this group displayed robust neutralizing antibody and T cell responses to the vaccine by 7 dpc. This lack of upregulation of important adaptive immunity genes in the Adt-O1M group provides further evidence of the adjuvanting capabilities of PEGboIFNλ3.

In conclusion, the current study is the first to demonstrate full protection of cattle against FMD conferred by administration of a recombinant, site-specific PEGylated bovine IFNλ3, and provides compelling rationale for applying this novel biotherapeutic in concert with FMD vaccines, as both an adjuvant as well as a means of bridging the gap in immunity during the first 3–7 days following vaccination.

## Data availability statement

The original contributions presented in the study are publicly available. This data can be found here: www.ncbi.nlm.nih.gov, accession number: GSE262192.

## Ethics statement

Ethical approval was not required for the studies on humans in accordance with the local legislation and institutional requirements because only commercially available established cell lines were used. The animal study was approved by Institutional Animal Care and Use Committee (IACUC) of the Plum Island Animal Disease Center (USDA/APHIS/AC Certificate number: 21-F-0001; Protocol 244.01-19-R). The study was conducted in accordance with the local legislation and institutional requirements.

## Author contributions

SaA: Conceptualization, Data curation, Formal analysis, Investigation, Methodology, Project administration, Resources, Supervision, Visualization, Writing – original draft, Writing – review & editing. CS: Investigation, Resources, Writing – review & editing. MR-C: Investigation, Resources, Writing – review & editing. AM: Investigation, Writing – review & editing. SoA: Investigation, Writing – review & editing. PA: Investigation, Writing – review & editing. PC: Conceptualization, Data curation, Formal analysis, Investigation, Methodology, Project administration, Supervision, Writing – review & editing. LS: Data curation, Formal analysis, Investigation, Methodology, Visualization, Writing – review & editing. JN: Data curation, Formal analysis, Investigation, Methodology, Visualization, Writing – review & editing. NK: Writing – review & editing, Conceptualization, Data curation, Formal analysis, Funding acquisition, Investigation, Methodology, Project administration, Resources, Supervision, Visualization. GM: Conceptualization, Investigation, Methodology, Project administration, Resources, Supervision, Visualization, Writing – review & editing. TS: Conceptualization, Funding acquisition, Methodology, Project administration, Resources, Supervision, Visualization, Writing – review & editing. FD-S: Conceptualization, Data curation, Formal analysis, Funding acquisition, Investigation, Methodology, Project administration, Resources, Supervision, Visualization, Writing – original draft, Writing – review & editing.
