# Supplementary material for: Prophylactic treatment with PEGylated bovine IFNλ3 effectively bridges the gap in vaccine-induced immunity against FMD in cattle
Source: Front Microbiol. 2024 Apr 4;15:1360397. doi: 10.3389/fmicb.2024.1360397 (PMC11024232; doi:10.3389/fmicb.2024.1360397)
Supplement: Supplementary file 1 [file Data_Sheet_1.DOCX]

Supplementary Material

# Supplementary Data

**Supplemental Table 1.** Summary results of work completed with animal #16, which moved during treatment and did not receive the full dose of PEGboIFNλ3.

| DPC | Antiviral Activity (units/ml serum) | Lymphocyte % of WBC | Clinical Score | Viremia (pfu/ml) | Viremia (GCN/ml) | Shedding (pfu/ml) | Shedding (GCN/ml) | Neutralizing Antibody Titer (Log_10_ Serum Dilution) | IFNγ+ % of CD4+ CD8- | IFNγ+ % of CD4-CD8+ | IFNγ+ % of CD3-CD8+ CD335- | IFNγ+ % of CD3-CD8+ CD335+ |
| --- | --- | --- | --- | --- | --- | --- | --- | --- | --- | --- | --- | --- |
| -5 | 0 | - | - | - | - | - | - | - | 0.2 | 0 | 2.28 | -2.9 |
| -4 | 41.84 | - | - | - | - | - | - | - | - | - | - | - |
| -3 | 37.34 | - | - | - | - | - | - | - | - | - | - | - |
| -2 | 29.84 | - | - | - | - | - | - | - | - | - | - | - |
| -1 | 0 | - | - | - | - | - | - | - | - | - | - | - |
| 0 | 0 | 64.02 | 0 | 0 | 0.0E+00 | 0 | 0 | 0 | 0.7 | -1.3 | -9.51 | 0 |
| 1 | - | 52.75 | - | 0 | 4.9E+03 | 10 | 0 | - | - | - | - | - |
| 2 | - | 58.88 | 0 | 0 | 0.0E+00 | 0 | 0 | - | - | - | - | - |
| 3 | - | 62.09 | 0 | 200 | 1.8E+05 | 190 | 0 | - | -1 | -0.7 | -3.68 | 1.45 |
| 4 | - | 50.55 | 1 | 925 | 8.2E+05 | 77.5 | 0 | 0 | - | - | - | - |
| 5 | - | 50.37 | - | 48 | 5.1E+04 | 0 | 0 | - | - | - | - | - |
| 6 | - | 49.55 | 3 | 0 | 0.0E+00 | 0 | 0 | - | - | - | - | - |
| 7 | - | 56.32 | - | 0 | 0.0E+00 | 1500 | 0 | 1.5 | 3.6 | 6.7 | 37.9 | 20 |
| 8 | - | - | 5 | 0 | 0 | 0 | 0 | - | - | - | - | - |
| 11 | - | - | 5 | - | - | - | - | - | - | - | - | - |
| 14 | - | - | - | - | - | - | - | 2.4 | 2.4 | 2.1 | 3.41 | 1.85 |
| 21 | - | - | - | - | - | - | - | 2.1 | 2.6 | 1.3 | 4.98 | 1.97 |
| 28 | - | - | - | - | - | - | - | 2.1 | - | - | - | - |

**
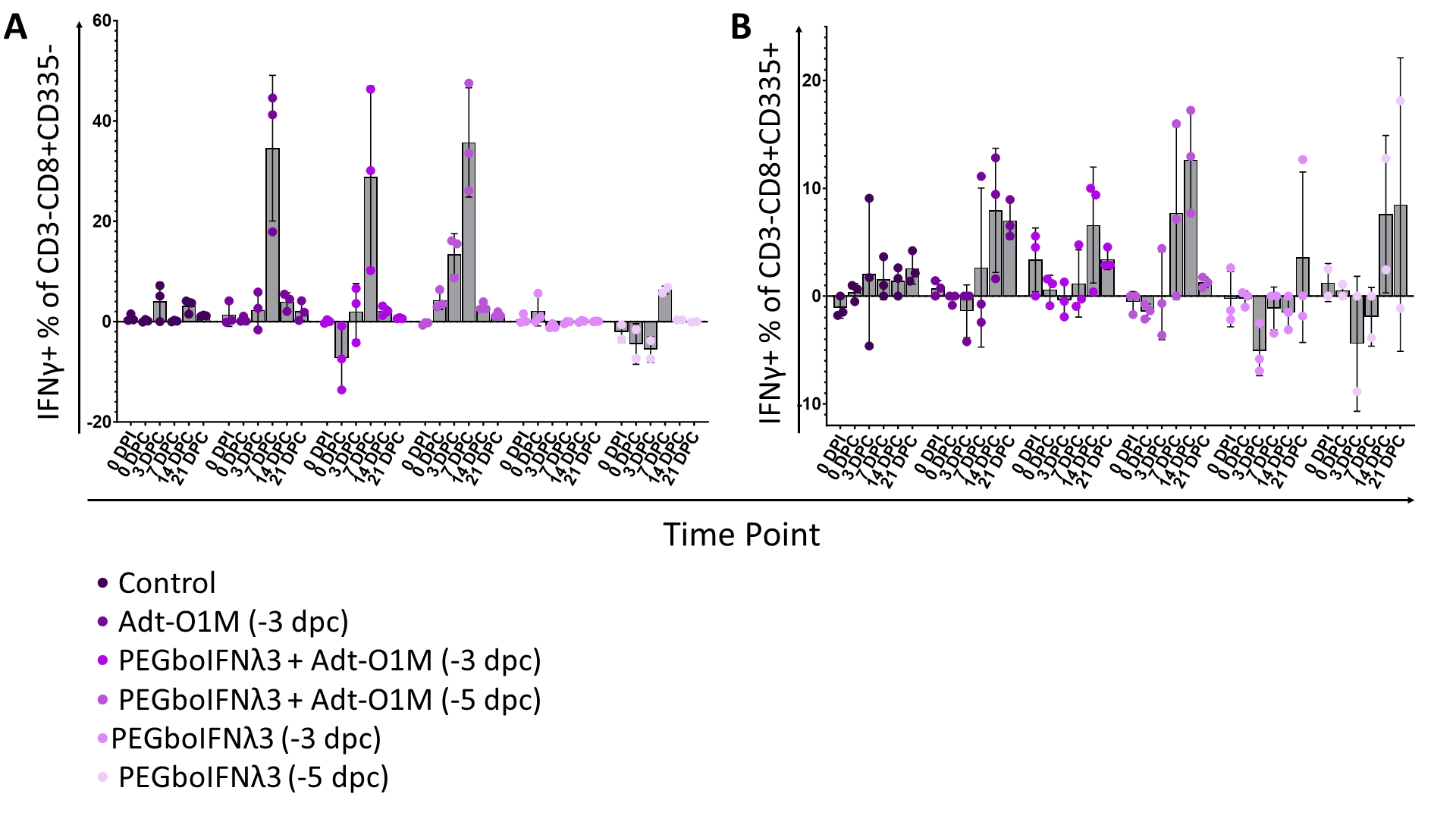
**

**Supplemental Figure 1.** Holstein calves of approximately 450 lb were inoculated SC with 150 μg/kg PEGboIFNλ3 and/or 2.5x10^9^ pfu Adt-O1M FMD vaccine at either 3 or 5 days prior to intranasopharyngeal challenge with 2x10^6^ BID_50_ FMDV O1Manisa. A control group was inoculated 3 days prior to challenge with 2.5x10^9^ pfu Ad5-Blue. Blood was collected daily after inoculation and challenge and PBMCs were isolated from whole blood, stimulated overnight with MOI2 of FMDV O1M and labeled for flow cytometric analysis and intracellular cytokine staining of IFNγ. Induction of in **A)** CD3-CD8+CD335- IFNγ+ **B)** CD3-CD8+CD335+ IFNγ+ as a percentage of the parent population were assessed. n=2-3 cattle/timepoint/treatment group.
